# Comparing timeliness, content, and disease severity of formal and informal source outbreak reporting

**DOI:** 10.1186/s12879-015-0885-0

**Published:** 2015-03-20

**Authors:** Chi Y Bahk, David A Scales, Sumiko R Mekaru, John S Brownstein, Clark C Freifeld

**Affiliations:** Boston Children’s Hospital, Informatics Program, 1 Autumn St, Boston, MA 02215 USA

**Keywords:** Disease outbreaks, Surveillance, Disease notification

## Abstract

**Background:**

Infectious disease surveillance has recently seen many changes including rapid growth of informal surveillance, acting both as competitor and a facilitator to traditional surveillance, as well as the implementation of the revised International Health Regulations. The present study aims to compare outbreak reporting by formal and informal sources given such changes in the field.

**Methods:**

111 outbreaks identified from June to December 2012 were studied using first formal source report and first informal source report collected by HealthMap, an automated and curated aggregator of data sources for infectious disease surveillance. The outbreak reports were compared for timeliness, reported content, and disease severity.

**Results:**

Formal source reports lagged behind informal source reports by a median of 1.26 days (p = 0.002). In 61% of the outbreaks studied, the same information was reported in the initial formal and informal reports. Disease severity had no significant effect on timeliness of reporting.

**Conclusion:**

The findings suggest that recent changes in the field of surveillance improved formal source reporting, particularly in the dimension of timeliness. Still, informal sources were found to report slightly faster and with accurate information. This study emphasizes the importance of utilizing both formal and informal sources for timely and accurate infectious disease outbreak surveillance.

## Background

Traditional infectious disease surveillance most often relies on cases recorded at healthcare facilities and diagnostic lab results, which are hierarchically reported to local, state, and national health authorities [1]. Such methods of surveillance, which historically have been characteristic of government or government-affiliated agencies, are prone to missing cases and time lags [1,2]. To overcome these limitations, many informal platforms, defined as surveillance incorporating data sources outside of government and clinical systems, have been developed in the last two decades. These include but are not limited to: BioCaster, Global Public Health Intelligence Network (GPHIN), Health Emergency Disease Information System (HEDIS), HealthMap, Medical Information System (MedISys), Pattern-based Understanding and Learning System (PULS), and Program for Monitoring Emerging Infectious Diseases (ProMED-Mail) [3,4].

In addition to the growth of informal surveillance platforms, the World Health Organization (WHO) revised the International Health Regulations (IHR) in 2005, changing the landscape of modern infectious disease surveillance [5-7]. The revised IHR instated a legal framework and procedure for outbreak detection, assessment, and reporting, putting pressure on government sources to rapidly report public health events [5-7]. Specifically, the revision requires governments to develop and maintain surveillance capacities in addition to the existing border screening requirements, to report events of possible concern to the WHO within 24 hours, and also explicitly allows the WHO to use non-governmental sources for outbreak intelligence [5-7].

These combined changes fostered a narrowing gap between formal and informal surveillance. For example, as ministries of health are building core capacities in surveillance and reporting as stipulated by the IHR revisions, it is expected that surveillance data be communicated in a more timely and transparent fashion [5,8]. Simultaneously, as informal surveillance efforts are growing and their value validated, informal surveillance data is increasingly being accessed and utilized by formal surveillance organizations, also exemplified explicitly by the IHR revisions [5,6]. As lines are blurred between formal and informal surveillance, their characteristics may be changing as well. For example, formal source reports have been expected and shown to be slower than informal sources but given greater value as the gold standard [1,8,9]. Another characteristic is that for severe diseases with potential political or economic impacts, formal source reporting may be biased and less transparent [8,10]. It can be hypothesized that with changes in the field, such differences between formal and informal source reports become less distinct. To test this hypothesis, initial outbreak reports from formal sources and informal sources were compared in timeliness, reported information, and disease severity.

Previous studies have compared timeliness between formal and informal sources, but have used a historic time frame of five to 20 years. The 2010 study by Chan et al. studied outbreaks from 1996 to 2006 and documented a 16-day lag between first informal communication of an outbreak and WHO Outbreak News [9]. Mondor et al. reported that government sources lagged 10 days behind non-government sources from 1996 to 2009 [1]. Tsai et al. reported in 2013 a 4.09 day lag between ProMED-Mail and WHO reports on avian influenza and H1N1 outbreaks between 2003 and 2009 [8]. Unlike these previous studies, the present study analyzes a more recent and narrow timeframe of six months in 2012, for an updated timeliness finding that is more relevant to current biosurveillance practices. This study also provides an additional data point to the downward trend observed in previous studies of the shortening time lag between formal and informal sources.

Analyzing the content of reports along with timeliness also differentiates this study. A timely report is valuable only with accurate information, and a later report would be expected to contain more information as the outbreak progresses with time. While previous timeliness studies compare initial outbreak reports, the potential difference in content of these reports is not analyzed [1,8,9]. In the present study, both variables are jointly analyzed to assess the relationship between timeliness and reported information.

Finally, outbreaks of severe diseases, which are more likely to impact trade or travel, are thought to be most prone to delayed reporting by formal sources due to potential political and economical consequences [8,11]. To the authors’ knowledge, no study to date has compared the effect of disease severity on the reporting behavior of formal and informal sources.

## Methods

The present study uses data from HealthMap (www.healthmap.org), which aggregates more than 200,000 disparate data sources onto a user-friendly and freely available interactive map [12]. Through automated online querying every hour in fifteen different languages, followed by automated and manual filtering and tagging of Web-based reports, the system facilitates real-time visualizations of emerging public health threats [12]. A detailed description of the system can be found elsewhere [12].

From June 1 to Dec 31, 2012, initial outbreak reports by formal sources were manually identified through MoH+, a HealthMap feed developed to actively gather streamlined formal source communication. This included websites, Facebook pages, and Twitter accounts of ministries of health, related ministries (e.g., agriculture, rural development), government portals, government affiliated surveillance organizations (e.g., CDC, FDA), and relevant international governing bodies (e.g., UN, WHO). The feed was built using more than 700 RSS feeds and APIs, a method described in a previous publication [13].

Initial outbreak reports were defined as event-based reports for one or more cases of any infectious disease with no reference to a related previous outbreak. These outbreak reports were manually matched in informal sources using the HealthMap feeds described in Table 1. Outbreaks were matched by searching for the disease and country of outbreak and reviewing resulting HealthMap informal reports. These informal reports may be utilized by formal sources (for example, Google News reports could be utilized by government agencies) or be drawn from a formal source (ProMED report relays a government statement). For the purposes of comparing formal and informal initial reports within HealthMap, the definition of a formal source report was that collected by MoH+. If formal outbreak information was collected through an informal HealthMap feed rather than through MoH+ which was specifically designed to pick up official reports, this points to the disseminative feature of the informal source report.

**Table 1:** **HealthMap feeds of informal source outbreak reports**

**Feed**	**Description**
Google News	A commercial news aggregation service provided by Google
Arabic RSS Feeds	Aggregation of news media in the Arabic language
Wildlife Data Integration Network	A news feed from the Global Wildlife Disease News Map provided by the NBII-Wildlife Disease Information Node at the US Geological Survey
Baidu	A Chinese language commercial news aggregation service provided by Baidu, the number 1 search engine in China
Soso	A Chinese language commercial news aggregation service provided by the Chinese search engine Soso
ProMED	Program for Monitoring Emerging Diseases, a program of the International Society for Infectious Diseases
HM Community	Online articles or reports submitted by community members through the HealthMap website
Eyewitness Reports	Approved alerts made by community members through HealthMap’s Outbreaks Near Me mobile application (healthmap.org/outbreaksnearme)

Of 170 unique initial outbreak reports in MoH+ during the study period, 111 (65.3%) were also reported by the informal feeds in Table 1. The 34.7% of outbreaks not reported by informal sources are a topic of interest in itself but were not the focus of the present study. The 111 outbreaks, with both a first formal report and a first informal report, constituted the final sample for analysis. A representative pair of reports is a BBC news article reporting seven cases of Legionnaires disease in Stoke-on-Trent, UK on July 24 2012 11:47 AM, and the UK Health Protection Agency reporting seven cases of Legionnaires’ disease in Stoke-on-Trent, UK on the same day 12:18 PM. Another typical example is a Marburg outbreak in Kibale, Uganda, a local news site reporting two cases on October 19 and the WHO reporting three cases on October 21.

For each outbreak, the issued date and time (standardized to Eastern Standard Time) of first report from MoH+ and from the informal source were identified, and the difference between the two was defined as the time lag. Following convention, the time lag was measured in terms of MoH+ lagging behind informal sources, with a positive lag signifying slower reporting by formal sources. To test for a significant difference in timeliness, the Wilcoxon sign rank test was performed to test the null hypothesis of no lag (0 seconds).

The content of each pair of first reports was manually reviewed and placed into the following categories: less information in MoH+ report, same information in both sources, or more information in MoH+ report. Most often, “more information” meant a higher case count or laboratory-confirmed cases rather than suspected cases, which were later verified to be accurate with subsequent reports. In the scenario of complete merging of formal and informal surveillance, the pair of initial reports would report the same information at the same time. The qualitative assessment of the information reported was intended to study the deviation from this scenario. The Kruskal-Wallis test was performed to test for significant difference in timeliness by information category.

Finally, to assess the effect of disease severity on timeliness and reported information, HealthMap’s disease severity scale of 1 to 5 was used. This scale measures the potential impact of the disease in social, economic, and political dimensions, with lower categories for routine and lower burden diseases and higher categories for very contagious, fatal, or high economic impact diseases. It was developed by a team of HealthMap analysts with extensive clinical, public health, and curation experience, well before this research was conducted. Although subjective and yet to be robustly validated, the majority of the US, UK, and Canadian notifiable diseases as well as the CDC Bioterrorism and Global Early Warning System’s priority diseases are 4’s and 5’s on the HealthMap disease severity scale (53%, 71%, 61%, 68%, and 74% respectively), suggesting that the scale mirrors potential severity of these diseases’ outbreaks. In the absence of an externally validated disease severity scale, this internal scale was used. The Kruskal-Wallis test was performed to test for significant difference in timeliness by disease severity category, and Fisher’s exact test was performed to test for association between reported information and disease severity.

## Results

**Figure 1:**
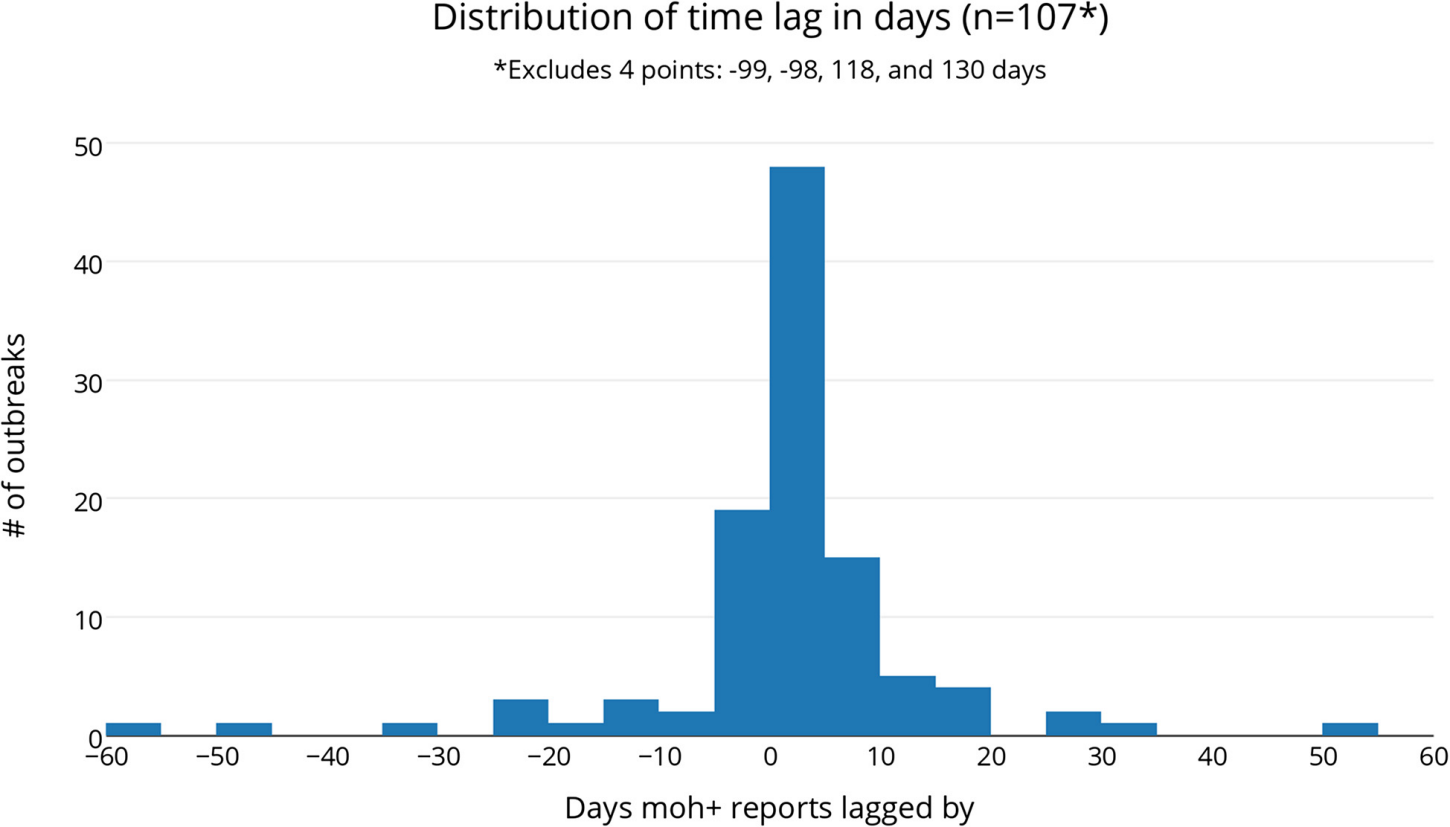
Distribution of time lag in days (n = 107*) *excludes 4 points: −99, −98, 118, 130 days.

Outbreak reports by formal sources lagged a median of 1.26 days (30.28 hours, p = 0.002). The full distribution can be seen in Figure 1. Of all outbreaks, 70% were reported first by informal sources and 30% first by formal sources. For 30% of outbreaks, first formal and informal reports were within 24 hours of each other. There were 20 outbreaks (18%) for which formal sources reported 24 hours or more before informal sources—10 by national, 9 by regional, 1 by international government sources. The majority of outbreaks (57%) fell in the 0 to 10 day lag category.

For 68 outbreaks (61%), there was “same information” in the first formal and informal reports. Among the 68, there was a significant lag (p = 0.003) in the timeliness of formal sources. Therefore, a majority of the time, formal sources lagged behind informal sources while reporting the same information. In the remaining 39% of outbreaks, formal sources reported more information in 22% and less information in 17%.

**Figure 2:**
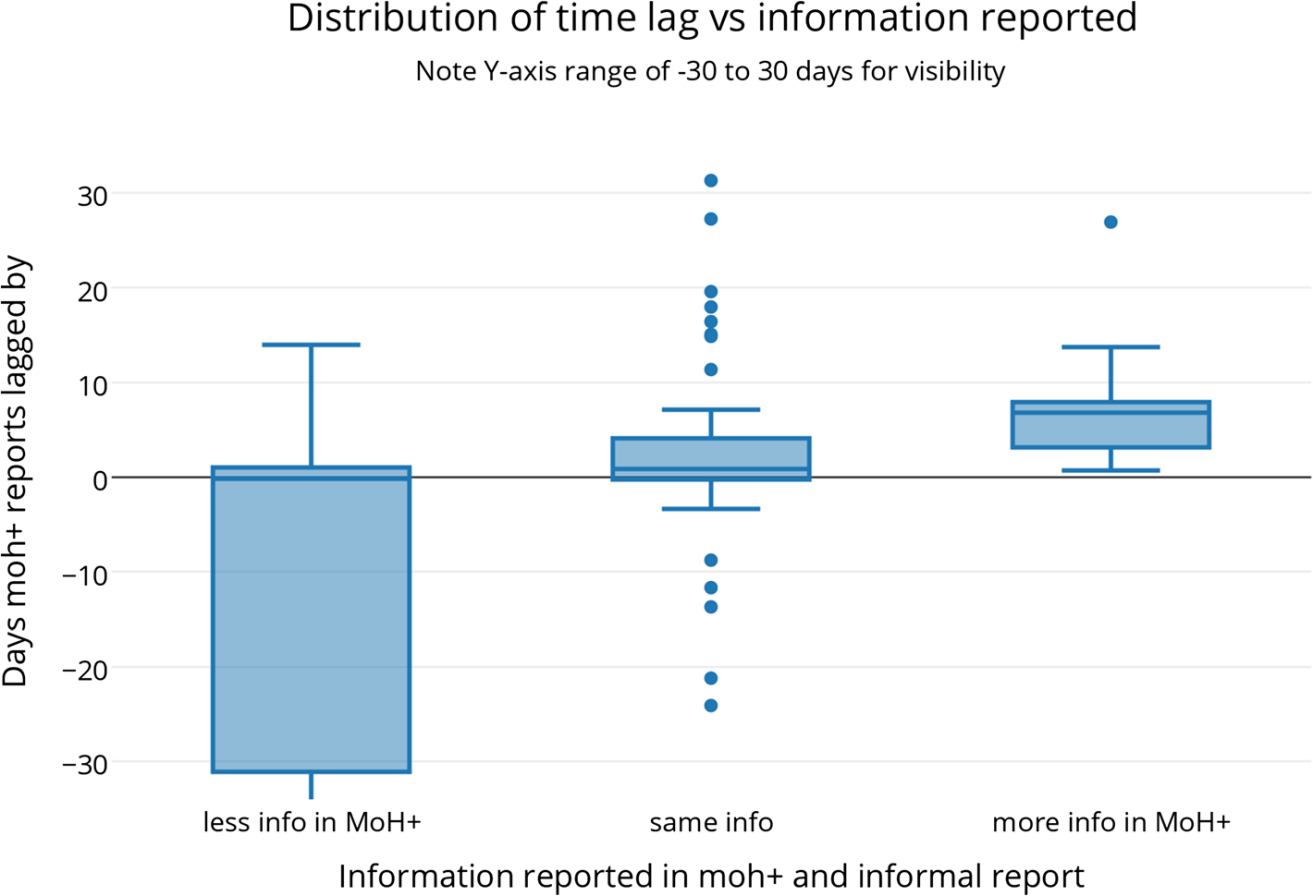
**Distribution of time lag vs information reported.**

Figure 2 shows the relationship between timeliness and reported information; the time lag was longest when the initial formal report provided more information (median lag = 6.78 days, p < 0.0001). In the other two categories, where MoH+ provides less or same information, the median lag was −0.21 and 0.85 days respectively (significant only in the same information category). Furthermore, the lag was significantly different among the three information categories (p = 0.0001), confirming the graphical trend that formal sources provide more information when they report later.

Taken together, the general trend for 89 outbreaks (80%) was two-pronged. In 59 (53%) of total outbreaks, reports from formal and informal sources reflect natural progression of the outbreak – formal and informal reports were published within 24 hours and contained same information, or if a formal report was available earlier by more than 24 hours, it contained less information and vice versa. In 30 outbreaks (27%), same information was reported between 1 to 51 days (median of 4.5 days) later by formal reports, as is conventionally explained by formal sources’ need for confirmation or buearocratic requirements.

The remaining were outbreaks that do not follow this trend. These included ten (9%) outbreaks for which formal sources reported on the same day or later with less information, possibly indicating that government agencies are not fully utilizing resources available to share outbreak information with the public -- although there may be valid reasons for witholding outbreak information, discussed below. Another 12 outbreaks (11%) were reported faster by formal sources and contained the same information, indicating government agencies have surveillance systems in place that ensure timely detection and reporting of disease.

In looking at disease severity, although the average severity was lower in outbreaks for which formal sources reported faster (3.58 versus 3.68), this observation did not pass the significance bar (p = 0.15).

## Discussion

The present study showed that from June to December 2012, HealthMap reports of formal sources lagged an average of 1.26 days behind informal sources, while the pairs of initial reports mostly reported the same information. This delay may stem from a desire to provide confirmed information or due to bureaucratic requirements. These findings suggest that informal sources provide early and accurate outbreak information, but also that formal sources have improved in speed and transparency compared to previous studies. These results also point to the closing gap between formal and informal sources, with majority of the pairs of initial reports containing the same information and the time lag continuously shortening.

Especially notable was the improved timeliness of formal sources compared to earlier studies reporting 16-day, 10-day, and 4-day lags and the continuous trend of shortening time lag [1,8,9]. Disease severity did not significantly impact relative timeliness of reporting, suggesting transparency in reporting diseases that may have political or economic impacts. Multiple factors may explain these findings, such as formal agencies’ increased adoption of online and streamlined communication, growth of informal surveillance as a competing source of information, or the 2005 IHR revision, which mandated and supported countries to practice more standardized formal surveillance and reporting.

Another possibility and a limitation of this study is that a system like MoH+ captures a distinct subset of formal sources that are more timely than formal sources overall, by being limited to formal sources that report online, are in a HealthMap-supported language, and have RSS feeds. Additionally, any online communication from a government-affiliated entity was included as a formal source report in MoH+, and a more restrictive definition of formal source reports could have been used. Nevertheless, the results suggest reporting online in a commonly supported language and using tools like RSS feeds and social media may allow formal surveillance organizations to disseminate reports faster through informal platforms like MoH+. Overall, these explanations point to the importance of collaboration between formal and informal surveillance systems to enable rapid dissemination of outbreak data.

Beyond timeliness, review of the reported information confirmed informal sources as a credible source of outbreak information. A pattern seen repeatedly was an outbreak report by an informal source (e.g. online news) followed by a formal source (e.g. the ministry) report the next day, or an informal source report of suspected cases versus a delayed formal source report of lab-confirmed cases. Disease severity analysis was inconclusive, suggesting formal source reporting was not delayed by disease severity in this dataset. Limitations in the content and disease severity analyses, respectively, were the use of the three content categories (less, same, and more information) making the study unable to capture more granular differences in information, and lack of a robustly validated disease severity scale. It would also be of interest to categorize reports further beyond formal and informal in future studies, to better characterize timeliness and content in specific types of sources.

Another limitation is the selected study outcomes – timeliness and content as often measured by case count – in that they may overlook other complexities in outbreak reporting, especially when looking only at the initial reports. However, it is reasonable that both timeliness and accurate case counts, albeit simplistic, are positive characteristics in outbreak reporting worthy of analysis over a large sample of outbreaks.

## Conclusion

This study showed that formal sources have improved in outbreak reporting practices, mainly in the dimension of timeliness. Informal sources, however, continue to provide insights slightly faster, most often reporting the same information as formal sources. These findings illustrate the merging of informal and formal surveillance, in that formal and informal sources are most often reporting the same information in closer time proximity than ever before.

The public health implication of these findings in the global context is that formal and informal surveillance can complement each other for more rapid and accurate outbreak reporting. The key for timely and accurate disease surveillance is not in relying on one type of source, but utilizing all possible sources in a way that is digestible to various stakeholders. With formal sources offering reliable, gold standard data from indicator based surveillance, and informal sources proactively collecting and disseminating event-based surveillance information, timely and accurate reporting can be achieved with existing tools.
